# Classification and occurrence of an abnormal breathing pattern during cardiopulmonary exercise testing in subjects with persistent symptoms following COVID‐19 disease

**DOI:** 10.14814/phy2.15197

**Published:** 2022-02-18

**Authors:** Anna von Gruenewaldt, Eva Nylander, Kristofer Hedman

**Affiliations:** ^1^ Department of Clinical Physiology in Linköping, and Department of Health, Medicine and Caring Sciences Linköping University Linköping Sweden

**Keywords:** cardiopulmonary exercise testing, COVID‐19, exercise capacity, hyperventilation, SARS‐CoV‐2

## Abstract

Reduced exercise capacity and several limiting symptoms during exercise have been reported following severe acute respiratory syndrome coronavirus‐2 (SARS‐CoV‐2) infection. From clinical observations, we hypothesized that an abnormal breathing pattern (BrP) during exercise may be common in these patients and related to reduced exercise capacity. We aimed to (a) evaluate a method to classify the BrP as normal/abnormal or borderline in terms of inter‐rater agreement; (b) determine the occurrence of an abnormal BrP in patients with post‐COVID; and (c) compare characteristics of post‐COVID patients with normal and abnormal BrP. In a retrospective, cross‐sectional study of patients referred for CPET due to post‐COVID April 2020–April 2021, we selected subjects without a history of intensive care and with available medical records. Three raters independently categorized patients’ BrP as normal, abnormal, or borderline, using four traditional CPET plots (respiratory exchange ratio, tidal volume over ventilation, ventilatory equivalent for oxygen, and ventilation over time). Out of 20 patients (11 male), 10 were categorized as having a normal, 7 an abnormal, and three a borderline BrP. Inter‐rater agreement was good (Fleiss’ kappa: 0.66 [0.66–0.67]). Subjects with an abnormal BrP had lower peak ventilation, lower exercise capacity, similar ventilatory efficiency and a similar level of dyspnea at peak exercise, as did subjects with a normal BrP. Patients’ BrP was possible to classify with good agreement between observers. A third of patients had an abnormal BrP, associated with lower exercise capacity, which could possibly explain exercise related symptoms in some patients with post‐COVID syndrome.

## Graphical abstract

A common finding in reports on patients with post‐COVID syndrome (or long COVID) is hyperventilation or disturbed breathing during exercise, while the definition of such a phenomenon or how frequent it occurs remains to be established. Using new criteria to define the breathing pattern during exercise as normal, abnormal, or borderline, we found that a third of patients with long COVID had an abnormal breathing pattern. The burden of patients with long COVID is expected to increase, and hereby the need fors a better understanding of how to interpret clinical findings at cardiopulmonary exercise testing, and how often they occur.

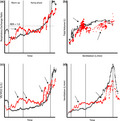

## 1 INTRODUCTION

It has become evident that a significant proportion of patients surviving a severe acute respiratory syndrome coronavirus‐2 (SARS‐CoV‐2) infection experience long‐term sequelae, often termed “long COVID” or post‐acute sequelae of severe acute respiratory syndrome (PACS) (Jiang et al., 2021; Lopez‐Leon et al., 2021) As also patients with only mild or moderate disease in the acute phase present with function limiting sequelae, this may potentially evolve into a huge worldwide medical problem (Naeije & Caravita, 2021). Although much effort has been made in understanding the mechanisms and epidemiology behind long COVID, the underlying pathophysiology remains elusive (Jiang et al., 2021).

The most common symptoms reported by patients with long COVID are fatigue and dyspnea. However, lung function tests have often failed to reveal a clear association between the degree of symptoms and objective findings in these patients (Froidure et al., 2021; Lerum et al., 2021). Therefore, cardiopulmonary exercise testing (CPET) has emerged as a method to quantify the level of impairment in exercise capacity, while also facilitating differential diagnostics (Naeije & Caravita, 2021). So far, no typical “long COVID pattern” at CPET has emerged, and a majority of studies report no specific cardiac or pulmonary reason for exercise intolerance (Barbagelata et al., 2021; Mohr et al., 2021; Skjorten et al., 2021). Interestingly, a few recent studies have reported signs of hyperventilation in patients with long COVID (Baratto et al., 2021; Motiejunaite et al., 2021; Singh et al., 2021).

In our clinical practice, we encountered patients with long COVID showing an irregular, and before the COVID‐19 pandemic unusual, breathing during CPET that did not fit into the usual pattern in exercise‐associated hyperventilation. We therefore, felt the need to characterize the breathing pattern (BrP) in a systematic manner, suggesting criteria for the observed irregularities, in order to describe the ventilation disturbances in these patients. The aims of the current work were to (a) classify the breathing pattern as normal/abnormal or borderline according to our suggested criteria in patients referred for CPET due to persistent symptoms following non‐critical COVID‐19 disease; (b) study the agreement of the classification by three independent observers; and (c) compare characteristics between long COVID patients with and without an abnormal BrP during CPET.

## 2 METHODS

### 2.1 Patients

In this cross‐sectional study, we retrospectively analyzed data from patients with persistent symptoms following COVID‐19 disease referred for CPET at the Department of Clinical Physiology, Linköping University Hospital in Linköping, Sweden from April 2020 to April 2021. Inclusion criteria were age > 18 years and a CPET performed ≥3 months following a PCR‐confirmed or a clinically very likely COVID‐19 disease (as PCR‐screening was uncommon in Sweden at the start of the pandemic). Exclusion criteria were COVID‐19 disease requiring intensive care or mechanical ventilation, underlying severe heart or lung disease, lack of full CPET data or no access to the patient's medical records. The study was approved by the Swedish Ethical Review Authority (no. 2021‐01620) and informed consent was waived for this retrospective analysis.

### 2.2 Patient background data

As patients were included retrospectively, data on patients’ symptoms, medical history, the severity of the acute phase of the COVID‐19 infection, and test results from SARS‐CoV‐2 PCR‐testing were retrieved from patients’ medical records. As per our exclusion criteria, patients where full access to medical records was not possible were not included.

### 2.3 Cardiopulmonary exercise testing

Following a dynamic spirometry including measurement of forced expiratory volume in one second (FEV1) and forced vital capacity (FVC), each subject underwent a maximal, symptom limited CPET on an electronically braked bicycle ergometer (eBike Basic, GE Medical Systems, GmbH, Freiburg, Germany). Gas exchange and ventilation was measured breath by breath (Vyntus CPX Carefusion, Hoechberg, Germany), with calibration of gas, pressure, and volume before each test. Each test was individually tailored, aiming at an exercise duration of 8–12 min, and started with 5 min at a submaximal steady‐state workload (10–50 Watts) followed by a continuous increment in workload of 10 or 20 Watts/min. During the test, the patient was continuously monitored with ECG while systolic blood pressure (SBP), rating of perceived exertion, dyspnea, and the occurrence and level of chest pain were rated every 2–3d min.

The percent of predicted peak oxygen uptake (VO_2peak_) was calculated using reference equations proposed by Gläser et al. (2010). The slope of ventilation (VE) over carbon dioxide (VCO_2_) elimination was determined graphically up until the respiratory compensation point using dedicated software (Sentry Suite v3.10), allowing for manual adjustment of the interval used for VE/VCO_2_‐slope calculation. The lowest 30 s mean value of VE/VCO_2_ was defined as the VE/VCO_2_‐nadir. By the default technical specifications of our CPET equipment, the ventilatory equivalent VCO_2_ (VE/VCO_2_) was corrected for mechanical dead space, while the VE/VCO_2_‐slope was not. Oxygen uptake, the VE/VCO_2_‐ratio, and end‐tidal CO_2_ partial pressure (PetCO_2_) was measured at the anaerobic threshold (AT), determined by two independent investigators using the V‐slope method in combination with the VE/VO_2_‐deflection point. In case of a difference in VO_2_ at measured AT > 10%, a third investigator was involved and the consensus was reached. Breathing reserve was calculated in relation to predicted maximal voluntary ventilation, multiplying FEV1 with 40. Predicted peak heart rate (HR) was calculated as 220‐age, predicted peak systolic blood pressure (SBT) and the SBP/Watt‐slope was calculated using published reference equations (Hedman et al., 2021).

### 2.4 Breathing pattern

**FIGURE 1:**
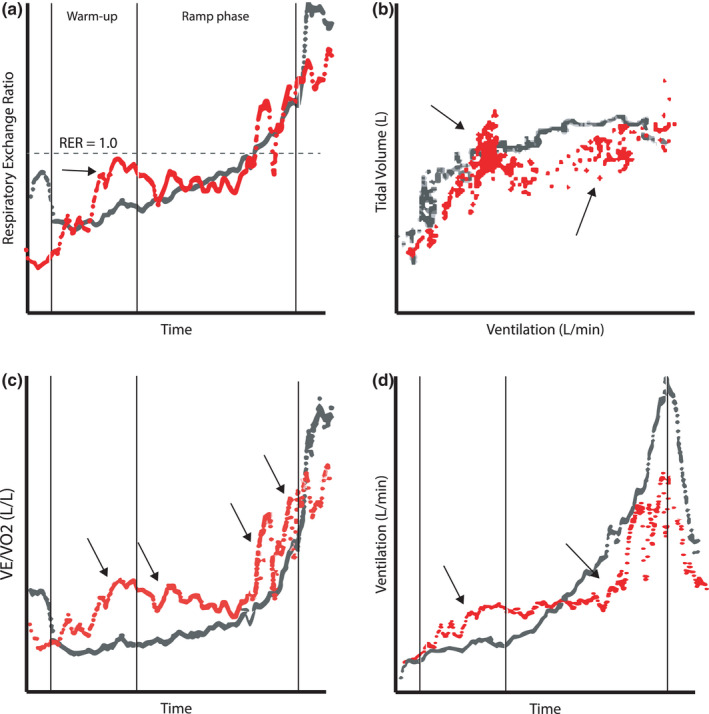
Ventilatory data in relation to the four criteria used to determine presence of an abnormal breathing pattern during exercise from two post‐COVID patients with normal (grey) and abnormal (red) breathing pattern. Arrows indicate points categorized as “abnormal” in each curve. Panel (a) shows the “overshoot” in the respiratory exchange ratio (RER) at submaximal exercise intensities (note that the abnormal increase in RER does not occur at the very start of exercise); Panel (b) shows the pattern of fluctuations in tidal volume at similar ventilation during exercise; Panel (c) shows the fluctuations of ventilation (VE) in relation to oxygen uptake (VO_2_) at several points during exercise and Panel (d) shows the unmotivated increases in ventilation during exercise below the respiratory compensation point. For Panel (c and d), this is different from the more regular and cyclic oscillations seen in severe heart failure (exercise‐induced oscillating ventilation, EOV)

Based on our initial clinical experience from the patients with long COVID, we decided a priori to categorize patients’ BrP as either “normal,” “abnormal,” or “borderline,” as clinical interpretation in some cases was difficult. Based on preliminary findings and following discussions among the authors, we used the following four criteria (see Figure 1): (a) an increase in the respiratory exchange ratio (RER) during submaximal steady‐state exercise or warm‐up, often after a few minutes of exercise (“RER overshoot,” in contrast to the common anxiety‐driven RER increase before and at the very beginning of exercise), followed by a decrease in RER during ramp exercise; (b) a fluctuation in tidal volume at the same minute ventilation (often paralleled by a corresponding fluctuation in breathing frequency); (c) an abnormal fluctuation in the ventilatory equivalent for oxygen (VE/VO_2_) during exercise; and (d) a sudden, unmotivated increase in ventilation during exercise below the respiratory compensation point. Of note, each of the criteria is non‐specific for long COVID, but our experience was that they occurred more often in combination in this group of patients than observed in other patient groups.

The occurrence of each criterion in each patient was determined independently by three separate investigators. If at least two out of four criteria were fulfilled, the investigator noted an “abnormal” BrP for that patient, while fulfillment of only one criterion was defined as “borderline.” In cases where all three investigators’ independent categorizations were not unanimous, the consensus was made through discussion.

### 2.5 Statistics

All data are presented as median with 25th to 75th percentiles, and Mann–Whitney *U*‐test or chi‐squared test were used to determine statistical significance in differences in distributions between groups. A two‐sided *p*‐value ≤ 0.05 was considered statistically significant. Inter‐rater agreement of BrP as normal/abnormal/borderline was assessed using Fleiss’ kappa. Analyses were carried out using SPSS statistics software version 27.0 (IBM corp., Armonk, NY, USA).

### 2.6 Patient and public involvement

Patients and/or the public were not involved in the design, conduct, reporting, or dissemination plans of this retrospective research.

## 3 RESULTS

### 3.1 Patients and symptoms

Out of 510 CPETs performed at the laboratory from April 2020 to April 2021, 27 (5%) were clinical referrals for follow‐up of patients with confirmed or clinically very likely COVID‐19. After excluding two tests being a second CPET in the same individual, two subjects with previous intensive care for COVID‐19, two subjects where medical records could not be accessed and one subject with <3 months from onset of acute COVID‐19 symptoms, CPET results from 20 subjects were included.

Of these 20 subjects (median age [IQR]: 47 [44–56], 11 male), 16 suffered from COVID‐19 during the first wave in Sweden (March 2020–June 2020) and four in November–December 2020. The median time from first symptoms to CPET was 31 weeks (IQR 19–47). While all four subjects from the second wave had a positive PCR test at the time of the acute infection, only 4/16 from the first wave (when testing was more uncommon in Sweden) had a positive test.

Eight subjects had been hospitalized during a median of 9 days (IQR 3–14 days), of which four had required high‐flow oxygen during hospitalization. The most common symptoms at the time of referral were dyspnea (*n* = 16, 80%), fatigue (*n* = 14, 70%), chest discomfort/pain (*n* = 13, 65%), difficulties with concentration (*n* = 8, 40%) and palpitations (*n* = 7, 35%).

### 3.2 Cardiopulmonary exercise testing

The median (IQR) percent of predicted FEV1 and FVC before exercise was 93% (87–107) and 89% (80–94), respectively, and the median FEV1/FVC‐ratio was 0.82 (0.78–0.86). All tests were terminated due to volitional fatigue, without any adverse event. No patient experienced a significant peripheral oxygen desaturation (<96%) during exercise. All except three subjects reached a RER > 1.10, and the median peak RER was 1.23 (1.14–1.30). The median breathing reserve was 31 (22–48), with two subjects having a breathing reserve <15% and/or <11 liters/min (7.8 and 14.7 liters/min, respectively).

The median peak VO_2_ was 26 ml/kg/min (21–31), or 94% (86–105) percent of predicted. Two patients (10%) had a peak VO_2_ below 80% of predicted. Median VE/VCO_2_‐nadir was 27 (25–30). In the 17 subjects where the anaerobic threshold was determinable, median VE/VCO_2@AT_ was 27 (25–31). Four subjects (24%) had a VE/VCO_2_‐nadir ≥30, while no subject had a VE/VCO_2_‐nadir over 35.

#### 3.2.1 Breathing pattern

**FIGURE 2:**
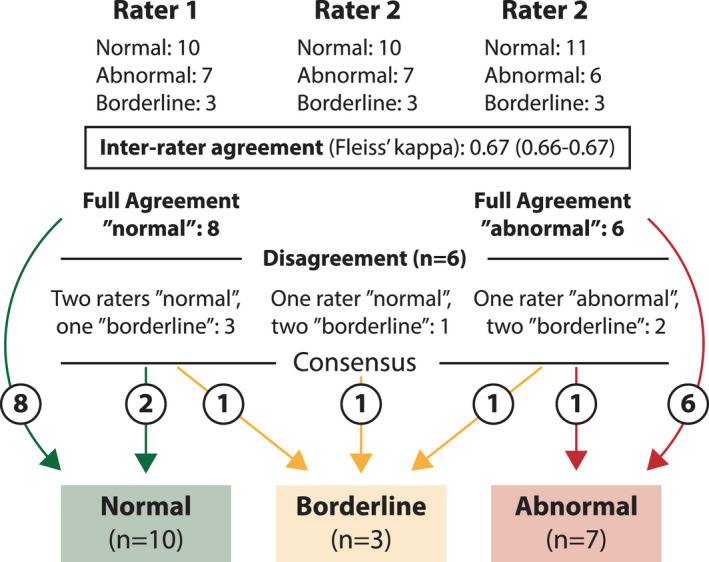
Categorization of breathing pattern in 20 subjects with previous COVID‐19 and current symptoms. For the underlying methodology used to define each pattern, see Figure 1 and “Section 2”

At the initial, independent categorization of patients’ BrP, a full agreement between the three investigators was obtained in 14/20 (70%) of patients (8 normal, 6 abnormal; Figure 2). Inter‐rater agreement was good for the initial, blinded overall classification of BrP (Fleiss’ kappa: 0.67, 95% CI 0.66–0.67). The inter‐rater agreement for the respective criteria (Figure 1a–d) were: Criterion a (RER): 0.45 (0.45–0.46); Criterion b (tidal volume): 0.72 (0.72–0.73); criterion c (VE/VO_2_): 0.48 (0.48–0.49): Criterion d (ventilation): 0.77 (0.76–0.78). In the six subjects where full agreement during blinded categorization was not made, the disagreement was between “borderline” and “normal” or “abnormal,” respectively, and not in any case between “abnormal” or “normal.” Following consensus discussions, an additional two subjects were defined as having a normal BrP, and one additional as an abnormal BrP, while three subjects (15%) were categorized as borderline BrP. As patients in the borderline group were few, we focused our analysis on differences between the 17 subjects with normal or abnormal BrP.

Background characteristics as per normal/abnormal BrP is presented in Table 1. The FEV1/VC‐ratio was similar between groups (normal BrP: 0.79 [0.77–0.84] vs. abnormal BrP: 0.85 [0.77–0.90], *p* = 0.48). Percent of predicted FEV1 and percent of predicted VC was also similar between groups (normal BrP: 93 [88–106]% and 89 [84–95]%, respectively; abnormal BrP: 93 [84–100]% and 86 [69–101]%, respectively, both *p* > 0.6).

**TABLE 1:** Background characteristics as per normal/abnormal breathing pattern

	Normal BrP *n* = 10	Abnormal BrP *n* = 7	*p*‐value
Male sex, *n* (%)	8 (80%)	2 (29%)	0.06
Age, years	47 (37–61)	52 (45–57)	0.74
Height, cm	177 (169–187)	171 (169–174)	0.13
Weight, kg	81 (72–84)	77 (62–111)	0.96
BMI, kg/m^2^	24.5 (23.0–28.9)	26.3 (21.7–36.7)	0.48
BMI > 30, *n* (%)	2 (20%)	4 (43%)	0.31
Smoking status as per medical records, *n* (%)			
Never	8 (80%)	3 (43%)	0.10
Previous	1 (10%)	3 (43%)	
Current	0	0	
Unknown	1 (10%)	1 (14%)	—
Symptoms reported in medical records, *n* (%)			
Dyspnea	7 (70%)	6 (86%)	0.45
Fatigue	6 (60%)	5 (71%)	0.63
Palpitations	2 (20%)	5 (71%)	0.034
Chest pain	6 (60%)	4 (57%)	0.91
Comorbidities in medical records, *n* (%)			
Hypertension	2 (20%)	3 (43%)	0.12
Hyperlipidemia	0	1 (14%)	0.22
Diabetes mellitus	0	0	—
Asthma	2 (20%)	0	0.21
Treatment during COVID‐19 infection, *n* (%)			
Hospitalized	4 (40%)	3 (43%)	1.0
Length of stay, median days (range)	13 (4–31)	3 (2–6)	0.11
Oxygen therapy	3 (30%)	1 (14%)	0.60
Thrombolysis	0	0	—
Steroids	4 (40%)	2 (29%)	1.0
Heparin	3 (30%)	1 (14%)	0.60

Note

Data presented as median (25th–75th percentile) or number of subjects (percent).

Abbreviation: BMI, body mass index.

**FIGURE 3:**
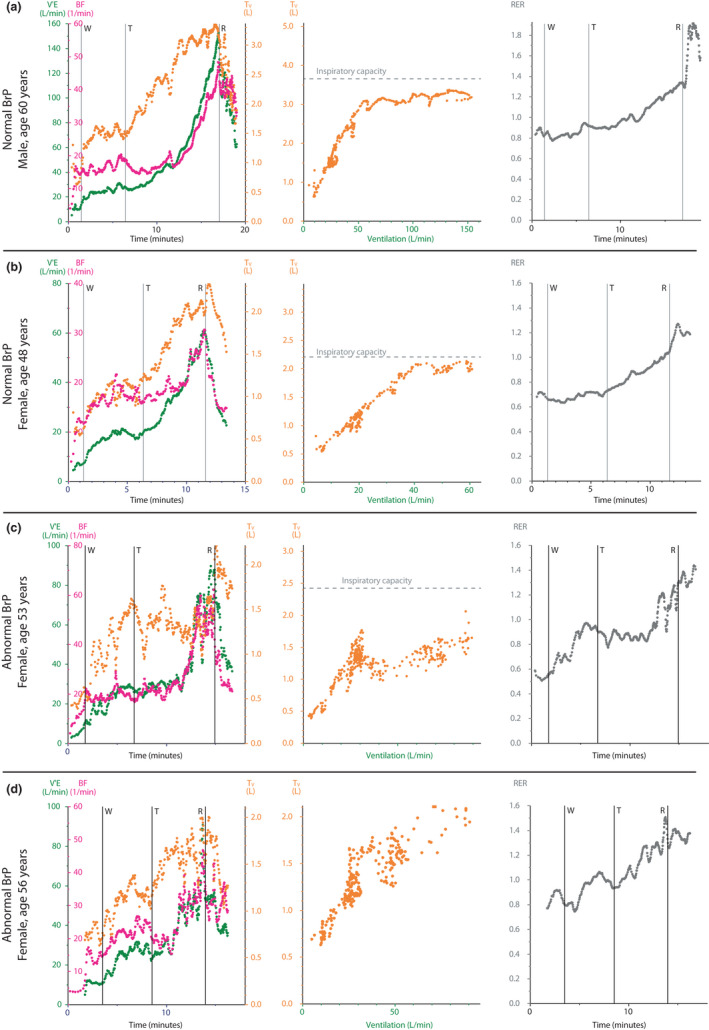
Selected ventilatory parameters during exercise from four patients with post‐acute COVID‐19 syndrome with or without an abnormal breathing pattern. Panel (a) and (b) exemplifies two subjects not categorized as having abnormal breathing (based on criteria presented in Figure 1), while Panel (c) and (d) present data from two subjects categorized as having an abnormal breathing pattern. BF, breathing frequency; BrP, breathing pattern; RER, respiratory exchange ratio; T_V_, expiratory tidal volume; VE, minute ventilation

Subjects with an abnormal BrP on average reached a peak workload of 110 Watts (94–117), versus 218 Watts (150–245) in subjects with a normal BrP. After considering age, sex, and height, subjects with an abnormal BrP had a significantly lower percentage of predicted peak workload than those with a normal BrP; 65% (60–73) versus 90% (79–115), *p* = 0.010. Ventilatory data from steady‐state and peak exercise during the CPET is presented in Table 2, and additional data from the CPET is presented in Table 3. Subjects with an abnormal BrP had lower ventilation at peak exercise (*p* = 0.019), due to a smaller tidal volume with similar breathing frequency, and a trend towards larger ventilatory reserve (*p* = 0.19). However, peak RER and rating of perceived exertion were similar between groups. The VE/VCO_2_‐nadir was similar in subjects with a normal and an abnormal BrP; 27.2 (24.7–30.7) versus 27.7 (26.2–29.4), *p* = 0.81, respectively. Typical patterns of breathing frequency, tidal volume, and ventilation during exercise in two subjects with and two subjects without abnormal BrP are presented in Figure 3.

**TABLE 2:** Ventilatory parameters during steady state and peak exercise

	State state/Warm‐up		*p*	Peak effort		*p*
	Normal BrP	Abnormal BrP		Normal BrP	Abnormal BrP	
VE, l/min	23 (21–27)	27 (23–44)	0.11	107 (75–122)	76 (59–78)	0.019
VE_reserve_, %	84 (82–86)	74 (70–79)	0.005	24 (17–44)	46 (29–50)	0.19
Vt, l	1.2 (1.1–1.7)	1.3 (1.2–1.5)	0.96	2.3 (2.0–2.9)	1.8 (1.6–2.1)	0.033
BF, 1/min	19 (15–25)	24 (19–36)	0.16	43 (37–49)	40 (35–47)	0.60
V_T_/IC	0.47 (0.34–0.50)	0.49 (0.41–0.55)	0.32	0.77 (0.61–0.89)	0.66 (0.61–0.74)	0.36
VE/VCO_2_	28 (26–32)	30 (27–35)	0.54	35 (32–39)	36 (31–43)	0.81
PetCO_2_, kPa	5.0 (4.5–5.3)	4.3 (3.7–4.7)	0.13	4.6 (4.0–5.2)	4.0 (3.4–4.8)	0.48
RER	0.85 (0.81–0.88)	0.97 (0.72–0.99)	0.13	1.25 (1.17–1.31)	1.21 (1.02–1.35)	0.36

Abbreviations: BF, breathing frequency; IC, inspiratory capacity; PetCO_2_, end‐tidal partial pressure for carbon dioxide; RER, respiratory exchange ratio; VCO_2_, carbon dioxide elimination; VE, minute ventilation; VE_reserve_, ventilatory reserve, calculated as 100 × [(FEV1 × 40 − VE)/(FEV1 × 40)]; Vt, tidal volume.

**TABLE 3:** Data from cardiopulmonary exercise testing

	Normal BrP *n* = 10	Abnormal BrP *n* = 7	*p*‐value
Heart rate and blood pressure			
Heart rate, rest (mmHg)	72 (61–82)	82 (62–94)	0.60
SBP, rest (mmHg)	120 (119–133)	140 (125–160)	0.09
DBP, rest (mmHg)	80 (69–80)	85 (70–95)	0.23
Heart rate, peak (1/min)	169 (154–185)	169 (146–179)	0.54
% pred peak HR	100 (89–106)	98 (87–104)	0.60
SBP, peak (mmHg)	190 (179–213)	195 (180–220)	0.67
% pred peak SBP	99 (91–108)	104 (95–109)	0.47
SBP/Watt‐slope (mmHg/Watt)	0.31 (0.28–0.44)	0.65 (0.37–0.70)	0.06
% pred SBP/Watt‐slope	82 (65–106)	129 (0.82–1.55)	0.09
HR recovery, 2 min (1/min)	−40 (34–46)	−33 (23–47)	0.48
HR recovery, 4 min (1/min)	−60 (54–70)	−48 (38–59)	0.60
Gas exchange parameters			
VO_2peak_ (ml/kg/min)	29.0 (24.6–32.9)	20.4 (15.6–23.5)	0.005
% pred VO_2peak_	96 (90–111)	85 (81–93)	0.043
VO_2@AT_, % of VO_2peak_	57 (53–71)	70 (62–72)	0.30
VO_2_/Watt‐slope (ml/min/Watt)	9.3 (9.1–9.8)	8.7 (8.6–9.9)	0.23
Oxygen pulse, peak (ml/beat)	14.6 (10.1–15.4)	8.5 (7.6–13.8)	0.06
Subjective measures			
RPE (6–20)	18 (17–19)	19 (17–19)	0.81
Dyspnea (1–10)	7 (6–8)	7 (6–7)	0.60
Chest pain (1–10)	0 (0–2)	0 (0–2)	0.96

Note

Data presented as median (25th–75th percentile) unless otherwise noted.

Abbreviations: AT, anaerobic threshold; CO_2_, carbon dioxide; DBP, diastolic blood pressure; HR, heart rate; RPE, rating of perceived exertion; SBP, systolic blood pressure; VE, ventilation; VO_2_, oxygen uptake; VO_2peak_, peak oxygen uptake.

## 4 DISCUSSION

We were able to categorize the breathing pattern of patients with long COVID as normal/abnormal or borderline with good agreement. We found that a third (7/20) of these patients had an abnormal breathing pattern, while only two (10%) of subjects had a VO_2peak_ < 80% of predicted and no subject experienced desaturation during exercise. Moreover, subjects with an abnormal BrP on average had lower exercise capacity, lower peak ventilation, and a trend towards higher breathing reserve, but with similar RER and perceived exertion at peak exercise. These results indicate that abnormal ventilation during exercise may at least in part explain symptoms of exercise intolerance during exercise in some patients with long COVID.

No study so far of those presenting CPET data in patients with long COVID and symptoms related to exercise have pointed out a typical or common cause of exercise intolerance or symptoms in this group of patients. In contrast, most studies suggest deconditioning without a specific underlying cardiac or ventilatory cause to exercise limitation (Barbagelata et al., 2021; Mohr et al., 2021; Rinaldo, Mondoni, Parazzini, Pitari, et al., 2021 ; Skjorten et al., 2021). Somewhat dependent on what definition of reduced exercise capacity is used, many studies report a surprisingly normal exercise capacity of patients, considering the burden of symptoms (Alba et al., 2021; Barbagelata et al., 2021; Rinaldo, Mondoni, Parazzini, Baccelli, et al., 2021 ). Thus, other factors than a severely reduced exercise capacity per se may impact on patients’ symptoms of dyspnea and exercise intolerance.

One of the first studies applying CPET in subjects with persistent symptoms following mild COVID‐19 disease was published as a case series of eight patients in January 2021 by Motiejunaite et al. (2020). They suggested “exercise hyperventilation” to be a major feature explaining exercise intolerance in this group of patients, based on a high VE/VCO_2_‐ratio (>40) in five subjects, of which three had a mild respiratory alkalosis at peak exercise. The same group of researchers later published CPET data on 114 consecutive patients, reporting “inappropriate hyperventilation” in 24% of subjects, defined as either of a VE/VCO_2_‐slope > 40, increased ventilatory equivalents for CO_2_ and O_2_ or high ventilation at the AT in absence of a pulmonary or cardiac limitation to exercise (Motiejunaite et al., 2021). In the light of our findings, it is unclear if—and to what extent—an abnormal BrP defined by our method of classification would account for their findings. Interestingly, Singh et al. recently utilized invasive CPET in 10 subjects with post‐COVID and 10 control subjects (Singh et al., 2021), and found an increased chemo‐sensitivity in post‐COVID subjects, defined as higher VE/VCO_2_ at lower PaCO_2_ values at the AT. Similar findings were reported by Baratto et al. (2021). In summary, it is possible that a typical hyperventilation, with respiratory alkalosis and disturbed chemo‐sensitivity, is present in a subset of patients with long COVID, whereas an abnormal BrP is a broader phenomenon that is multifactorial and more prevalent. Whether these subjects would benefit from physical therapy focused on restoration of a normal breathing pattern to the same extent remains to be elucidated.

While our results remain to be validated in a different cohort of patients, and compared to findings in a control group, in our clinical experience, the prevalence of this type of abnormal BrP is significantly more common in long COVID patients than in other patient groups undergoing CPET. In contrast to what is seen in transient hyperventilation at the start of exercise, the abnormal BrP seen in our subjects with long COVID included an abnormal overshoot in RER later during the steady‐state warm‐up phase, often starting at minute two or three. In addition, a majority of subjects with abnormal BrP had unusual fluctuations in ventilatory patterns across the whole exercise test, including a rise in ventilatory equivalents during the last minutes of exercise, which is not commonly seen in transient hyperventilation.

We found that subjects with an abnormal BrP had a lower exercise capacity than those with a normal BrP, but they did not have any evidence of a ventilatory limitation to exercise. In contrast, they reached lower peak ventilation and had a tendency of having a larger breathing reserve (46 vs. 24%, *p* = 0.19), with similar RER and ratings of dyspnea and perceived exertion at peak exercise. In addition, measures of ventilatory efficiency were similar between groups. Thus, it seems unlikely that the abnormal BrP could be attributed to a ventilatory constraint or pathology. The oxygen pulse (VO_2_/HR) at peak exercise was lower (*p* = 0.06) in subjects with an abnormal BrP, which could be taken as an argument for cardiac dysfunction, as the oxygen pulse is in part a surrogate for stroke volume. However, subjects in the abnormal BrP group were more often female and had a trend of being of lower height, which could explain part of the difference in oxygen pulse. And, importantly, the pronounced variations in VO_2_ due to the abnormal BrP make any interpretation of the oxygen pulse (as well as of the VO_2_/Watt‐slope) difficult. It is possible that an abnormal BrP in this group of patients is linked to an imbalance or dysfunction of the autonomous nervous system, similar to what has been reported regarding cardiac dysautonomia or postural tachycardia syndrome, POTS in post‐COVID patients (Goldstein, 2021). In favor of this speculation, we found a history of palpitations more common in subjects with an abnormal BrP than in those with a normal BrP (71 vs. 20%). The underlying pathophysiological mechanisms of a potential autonomic dysfunction remain largely unknown (Jiang et al., 2021).

Of note, the proportion of subjects with abnormal values in the VE/VCO_2_‐nadir and VE/VCO_2_‐slope varied depending on what measure of ventilatory efficiency was used. This may be of particular importance in situations where the scientific community is struggling to understand a new disease entity, and initial findings are divergent. Indeed, several studies have reported higher VE/VCO_2_‐slope values in patients with long COVID compared to control subjects (Baratto et al., 2021; Raman et al., 2021; Singh et al., 2021), while others have found similar VE/VCO_2@AT_ (Szekely et al., 2021) or VE/VCO_2_‐nadir (Alba et al., 2021). However, comparisons between studies should be made with caution as there are differences in the methodology used in the determination of the VE/VCO_2_ relationship, and whether mechanical dead space is subtracted from each measurement of ventilatory efficiency is rarely specified. Moreover, different approaches may be used by different CPET vendors, and our equipment does subtract mechanical dead space from the VE/VCO_2_‐nadir, while including mechanical dead space in the VE/VCO_2_‐slope, making direct comparisons between these measures impossible.

### 4.1 Limitations

First, the sample size of this exploratory study was small, limiting the power to detect differences between groups based on BrP. Second, the lack of a control group precludes any comparisons in terms of BrP with healthy subjects or patients with other cardiopulmonary conditions, which would be of particular interest. Third, we relied on data on symptoms from patients’ medical records rather than questionnaires, which could give a more accurate and in‐depth description of current symptomatology. Finally, not all subjects had a PCR‐verified diagnosis of COVID‐19, due to the relatively low number of PCR‐tests during the first wave of the pandemic in Sweden. Nevertheless, in cases without verified disease, we carefully selected patients where the referring doctor had strong reasons to suspect an underlying COVID‐19 infection as cause of the patient's symptoms.

## 5 CONCLUSIONS

Abnormal breathing during exercise was common in subjects with long COVID referred for CPET and could theoretically explain some of the symptoms these patients experience during exercise. Our method of classification of breathing patterns needs validation in larger cohorts with different pathological etiology, and could possibly guide the need for specific rehabilitation focused on restoring a normal breathing pattern.

## CONFLICT OF INTEREST

None.

## AUTHOR CONTRIBUTION

All authors participated in project planning. AvG and KH measured and analyzed CPET data. All authors were engaged in test‐retest analysis of breathing patterns. AvG retrieved data from patient records. KH performed statistical analysis. AvG and KH drafted the first version of the manuscript, and all authors critically revised the manuscript into its final form.

## ETHICAL APPROVAL

The study was approved by the Swedish Ethical Review Authority (no. 2021‐01620).
